# WikiHyperGlossary (WHG): an information literacy technology for chemistry documents

**DOI:** 10.1186/s13321-015-0073-7

**Published:** 2015-05-22

**Authors:** Michael A Bauer, Daniel Berleant, Andrew P Cornell, Robert E Belford

**Affiliations:** Myeloma Institute for Research and Therapy, University of Arkansas for Medical Sciences, 4301 West Markham, Slot 776, Little Rock, AR 72205 USA; Department of Information Science, University of Arkansas at Little Rock, 2801 S. University Avenue, Little Rock, AR 72204 USA; Department of Chemistry, University of Arkansas at Little Rock, 2801 S. University Avenue, Little Rock, AR 72204 USA

**Keywords:** Glossary, Hyperglossary, Semantic web, Social web, Information literacy, Perl javaScript, Gold book

## Abstract

**Background:**

The WikiHyperGlossary is an information literacy technology that was created to enhance reading comprehension of documents by connecting them to socially generated multimedia definitions as well as semantically relevant data. The WikiHyperGlossary enhances reading comprehension by using the lexicon of a discipline to generate dynamic links in a document to external resources that can provide implicit information the document did not explicitly provide. Currently, the most common method to acquire additional information when reading a document is to access a search engine and browse the web. This may lead to skimming of multiple documents with the novice actually never returning to the original document of interest. The WikiHyperGlossary automatically brings information to the user within the current document they are reading, enhancing the potential for deeper document understanding.

**Results:**

The WikiHyperGlossary allows users to submit a web URL or text to be processed against a chosen lexicon, returning the document with tagged terms. The selection of a tagged term results in the appearance of the WikiHyperGlossary Portlet containing a definition, and depending on the type of word, tabs to additional information and resources. Current types of content include multimedia enhanced definitions, ChemSpider query results, 3D molecular structures, and 2D editable structures connected to ChemSpider queries. Existing glossaries can be bulk uploaded, locked for editing and associated with multiple social generated definitions.

**Conclusion:**

The WikiHyperGlossary leverages both social and semantic web technologies to bring relevant information to a document. This can not only aid reading comprehension, but increases the users’ ability to obtain additional information within the document. We have demonstrated a molecular editor enabled knowledge framework that can result in a semantic web inductive reasoning process, and integration of the WikiHyperGlossary into other software technologies, like the Jikitou Biomedical Question and Answer system. Although this work was developed in the chemical sciences and took advantage of open science resources and initiatives, the technology is extensible to other knowledge domains. Through the DeepLit (Deeper Literacy: Connecting Documents to Data and Discourse) startup, we seek to extend WikiHyperGlossary technologies to other knowledge domains, and integrate them into other knowledge acquisition workflows.

**Electronic supplementary material:**

The online version of this article (doi:10.1186/s13321-015-0073-7) contains supplementary material, which is available to authorized users.

## Background

Jean-Claude Bradley was a pioneer in both open science and the application of social web technologies to chemical education. This paper describes an information literacy technology that was created for the chemical education community, the WikiHyperGlossary (WHG) [1]. This technology integrates hypertext with a variety of open science initiatives and technologies. The name WikiHyperGlossary reflects the initial goal of the project, which was to enhance reading comprehension of documents by connecting them to socially generated multimedia definitions. As the work progressed the scope of the project extended to a semantic web application that connects data to documents within the chemical sciences. This technology can be of value to both experts and novices and is extensible to other knowledge domains. Jean-Claude was an inspiration for many of us, he was present when the idea of this project first came about, and his creativity will be missed.

### Hypertext and 21^st^ century information literacy challenges

The United Nations considers literacy to be a fundamental human right [2]. This issue is of critical concern in nations and cultural contexts where segments of the population lack the fundamental literacy skills required to effectively participate in modern civilization. The World Wide Web has also created new literacy challenges for wealthier and more literate nations. Where today, even educated people have ready access to multitudes of documents they cannot comprehend.

The web is built on hypertext as a foundation. Hypertext is a concept, not a particular piece of software. However software implementations are what brought hypertext into widespread internet use. The first such implementation was called Gopher. Users would typically see a screen showing text, followed by a list of link targets to choose from by typing on the keyboard [3] (mice were not yet common). The World Wide Web (WWW) became publically available in 1991 and quickly grew to dominate the hypertext world. It was supported not only by a Gopher-like text-only browser that still exists, Lynx [4], but by browsers that could handle images and other multimedia information as well. This is the type of browser in common use today. With multimedia support the web made the leap from hypertext to hypermedia, and more quantum leaps in hypermedia technology followed.

One such leap was the invention of the search engine, a far more useful utility than the simple jump page. This enabled the web to serve as a comprehensive information resource, a digital library matching the vision put forth by H. G. Wells in his 1938 essay “World Brain” [5]. Another was the technology of social networking in its multitudinous implementations. As the world of reader interaction systems [6] progressed to still more advanced hypermedia systems, the link itself has become more sophisticated in concept and implementation. The common case of author-created and therefore static and explicit links can be extended to dynamic links by systems that suggest links to the author, or even automatically add them at the reader’s request. This can facilitate a high density of new links that can support a user experience approaching dialogues with documents [7].

There are several ways dynamic links can be added; such as by browser add-ons, software applications displaying the page, or processing a page through server side applications and viewing in a browser. Dynamic links may result from controlled vocabularies, where only specific words are linked, or uncontrolled vocabularies where every word is linked to new resources through a search engine or other information portal. The Hyperwords browser add-on [8] used an uncontrolled vocabulary that linked any word in the document to a variety of resources through a drop-down box. Although this add-on is no longer supported, the work continues with Liquid Words [9]. Examples of server side processing involving uncontrolled vocabularies would be the translation services like thai2english [10] and the WikiHyperGlossary’s JavaScript Automated Search (JAS) [11]. The former implemented mouse hover links that would pop up word translations that had been added to the document, and would work even if the returned document was disconnected from the web, while the latter was similar to HyperWords in that it sent highlighted words to different search services. Examples of controlled vocabularies would be the Utopia Document PDF reader [12], and the MSDS DeMystifier [13] of which the WikiHyperGlossary evolved out of. The former is a software application (PDF reader) while the later involves server side processing, that will be described in this paper. It could be argued that Utopia Docs also introduces another type of dynamic link, which is not at the text level, but the document level, and connects the entire document to material like related literature, altmetrics and citations. These are not linked text in the traditional sense, but appear in a side panel of the Utopia PDF reader and help the user relate an article and its authors to the broader scientific community. Another tool that has a similar functionality to one of the features of the WikiHyperGlossary is ChemAxon’s chemicalize.org [14] resource, which identifies chemical structures in documents and provides a search interface to identify molecules with similar structure. The WikiHyperGlossary also identifies chemicals in textual documents while allowing the 2D structure to be altered and additional information on that new structure acquired.

Publishers are also enabling dynamic links in published articles with server-side resources like ChemSpider Synthetic Pages [15] and Project Prospect of the Royal Society of Chemistry [16]. These enhance scholarly articles with supplementary information that supports the needs of readers. In fact the RSC has recently retired the name ‘Project Prospect’ as the approach is now integrated within their routine publication process ([16]). Articles supported by this enhanced publication environment appear in a Web browser as HTML documents that allow readers to activate and follow hyperlinks from terms in the article to information in ChemSpider [17], ChEBI [18], and the IUPAC Gold Book [19]. An overview of Project Prospect (and Utopia) can be found on YouTube [20]. A critical difference between publisher offered resources like Project Prospect, and ones like Utopia Docs, Liquid Words and the WikiHyperGlossary, is that the reader can submit documents of their choosing to the latter, while the former are only available for articles the publisher offers.

### Origins of the WikiHyperGlossary (WHG)

During the 2006 online ConfChem [21] conference Jean Claude Bradley presented the paper, “Expanding the role of the organic chemistry teacher through podcasting, screencasting, blogs, wikis and games” [22] the same week Toreki and Belford presented a paper on the MSDS HyperGlossary [23]. The MSDS HyperGlossary had a feature, the MSDS DeMystifier, that would automate the markup of MSDS (Material Safety Data Sheets), inserting links and connecting them to definitions within the MSDS HyperGlossary. Belford’s students would write definitions designed to enhance reading comprehension of MSDSs (whose target audience ranged from janitors and shop-room mechanics to PhDs), that were emailed to Toreki, who in turn uploaded them to the MSDS HyperGlossary. Rzepa [24] and Mader [25] also presented papers on wikis and during the ensuing discussions the idea of merging these two technologies came forth, which led to the concept of the WikiHyperGlossary (WHG).

Belford and Killingsworth created the first instance of the WHG that was demonstrated at the 2006 BCCE (Biennial Conference on Chemical Education) and presented in the Fall 2006 CCCE Newsletter [11]. Work continued with multilingual functionality and the IUPAC Gold book being integrated into the HyperGlossary as presented by Sullivan, et al. [26]. In 2009 NSF funding was received to develop a WikiHyperGlossary for the Chemical Education portal of the NSDL, ChemEd DL [27]. This led to the current work that we are reporting on, and there are currently two different instances of the WHG, the production site at ChemEd DL [28] which is maintained by the ACS Education Division, and the development site at hyperglossary.org, which is maintained by DeepLit and the authors of this paper.

The original vision of the WHG was of an information literacy technology to deal with one of the challenges of the web age, understanding documents in one’s distal knowledge space. Search engines can instantly provide access to expert-to-expert level documents that novice readers lack the background knowledge to understand. The inevitable consequence is shallow surface browsing through multiple documents until novices find comprehensible material at their level. This material may lack the veracity and accuracy of expert-to-expert level documents. E.D. Hirsch points out in the Knowledge Deficit [29], that reading comprehension not only requires understanding 90 % of the domain specific terms in a document, but also latent (implied) knowledge which the experts assumed readers possess. To quote E.D. Hirsch, “In fact what the text doesn’t say often far exceeds what it says” [29], and this leads to the crux of the problem. How do you provide the novice with the implied knowledge that the expert assumed the reader possessed when they wrote the expert-to-expert level document?

### Using chemical identifiers to couple open source applications and resources to documents

While developing the WikiHyperGlossary (WHG) for the Chemical Education Digital Library we came to realize that we were working with a unique class of words, the names of chemicals, for which we could assign chemical identifiers. We chose to use the InChI to handle this, opening a whole new dimension to the information content the WHG could provide. Our initial work took advantage of open-source communities like the Blue Obelisk [30], and through open source software applications like JChemPaint [31], Open Babel [32] and Jmol [33], we were able to populate chemical definitions with 2D and 3D molecular visualization software agents. The chemical identifiers also enabled us to connect both definitions and molecules created with the molecular editor to a plethora of chemical information sources through open access chemical compound data portals like ChemSpider [17] and PubChem [34]. When we moved to a cloud based service we started using the ChemSpider Open Babel API, and in 2014 removed all Java based software, changing Jmol to JSmol [35], and JChemPaint to the JSME editor [36]. Although this work was developed in the chemical sciences and took advantage of open science resources and initiatives, the technology is extensible to other knowledge domains. Information literacy technologies like the WHG can also be integrated into other software applications, and this paper will also report on the integration of the WHG into the Jikitou Biomedical Question and Answer System [37].

## Implementation

### WHG software architecture

The philosophy of open access data, open source software, and open standards was a driving force in the software architectural design decision for the WHG, an adaptive information literacy technology that is customizable to multiple contexts and domains. The leveraging of different open source tools and open access knowledge bases, while taking advantage of open standards, helped greatly in implementing the WHG application because they enabled pulling information from the wealth of expert knowledge in the community [38]. The WHG is also open source and hosted in a public repository on GitHub. Its core server side components are written in Perl and make extensive use of the Comprehensive Perl Archive Network (CPAN) [39], again taking advantage of open source resources by using Perl libraries written by the Perl programming community. The WHG is integrated with a MYSQL database backend. It can be deployed on a Linux distribution running an Apache web server. A detailed list of resources and tools used and integrated into the WHG is presented in Table 1. The WHG can be run on virtual or dedicated servers, and several options for accessing or running the WHG are presented in the Availability and Requirements section of this document.

**Table 1:** Detailed overview of resources and tools integrated into the WikiHyperGlossary system

Product	Description	Version
Software Architecture Tools		
Perl	General purpose programming language.	5.10.1
Catalyst	Model View Controller framework for the Perl language.	5.90007
ExtJS	JavaScript application framework for building interactive web applications.	3.x
MySQL	MySQL is an open source database management system.	14.12
Integrated Applications		
JSmol	JSmol extends the Java-based molecular visualization applet Jmol (jmol.sourceforge.net) to an HTML5 JavaScript-only web app.	14.0.2
JSME	JSME is a free molecule editor written in JavaScript.	2012-06-28
TinyMCE	TinyMCE is a platform independent web based Javascript HTML WYSIWYG editor module.	3.0
Balloon	Balloon creates 3D atomic coordinates from molecular connectivity data via distance geometry and conformer ensembles using a multi-objective genetic algorithm.	1.0.3.734
Web Services		
ChemEdDL Models 360	http://www.chemeddl.org/resources/models360/models.php	2014
ChemSpider	*ChemSpider* is a free chemical structure database with access to a range of web services. http://www.chemspider.com/	2014
	• SMILESToInChI (Convert Smiles to InChI)	
	• Convert (Uses OpenBabel Internally to convert)	
	• GetCompoundThumbnail	
	• SimpleSearch	
CACTUS	This service resolves different chemical structure identifiers and allows converting a given structure identifier into another representation or structure identifier. http://cactus.nci.nih.gov/chemical/structure	13th December 2014 14:13

**Fig. 1:**
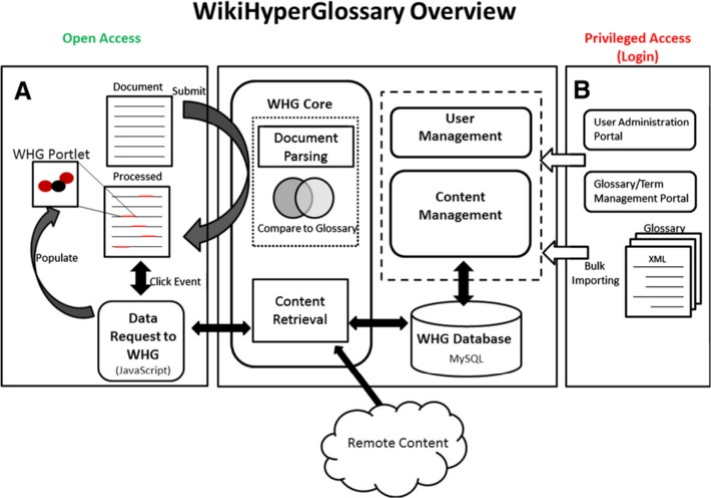
An overview of the main components of the WHG system. **a** The process of submitting a document to the WHG. Once a document is processed it contains elements that allow it to interact with the WHG server to pull information into the WHG Portlet. The portlet appears when tagged terms (distinguished by a different color) are selected. **b** The components that allow authorized users to manage the WHG content and users. Tools and functions have been created that aid the creating and importing of glossaries

The WHG software architecture is divided into two distinct functional components: WHG Core and Content Management (for user administration and glossary management). The WHG Core component does document processing, which automates the markup of text documents, linking them to material that is displayed in a JavaScript overlay, the WHG Portlet (see Fig. 1). The Content Management functions require login and vary by user group. No login is required for the public user interface that allows the user to submit documents for processing and interact with the processed pages that can call information through the WHG Portlet.

### WHG core: linking to semantically relevant content

A key feature of the WikiHyperGlossary is its ability to enable users to automate the hyperlinking of words in documents to data and definitions in a glossary of their choice. A user reading a processed document can click a linked term and conveniently retrieve additional pertinent content without having to leave the document. The system thus uses a chosen glossary to connect traditional textual information to dedicated knowledge associated with the lexicon’s domain. This provides relevant information to support understanding and knowledge exploration in domains of the reader’s choice.

An overview of document processing and knowledge retrieval functionality is shown in Fig. 1. A source URL or pasted text is submitted through the web interface and the glossary, which corresponds to a specific domain, is chosen. The document is processed using regular expression matching to identify strings comprising words and phrases germane to the particular domain. Strings that are matched are replaced with HTML span tags, which we refer to as HG tags.

$$ < span\  class=" hg3"\  context=" search"\  wordterm=" ozone">\mathbf{ozone}</ span> $$

The HG tags are used to register click events with a JavaScript function that asynchronously sends the term being clicked to the WHG server and waits for content to be returned. Some documents such as web pages have pre-existing links that need to be preserved. This is done by segregating documents into “safe” and “unsafe” portions. Unsafe portions contain pre-existing links and script tags that are left unprocessed, and safe portions are sent on to be parsed and modified with the HG tags. The safe and unsafe segments are then reassembled and the page is returned to the browser. The resulting processed document contains hyperlinked key words and phrases shown in a different color, typically green, differentiating them from pre-existing links. When a user clicks on one of these HG tagged terms the information associated with the term is retrieved and displayed in the WHG Portlet, which is superimposed on the document.

### WHG portlet

**Fig. 2:**
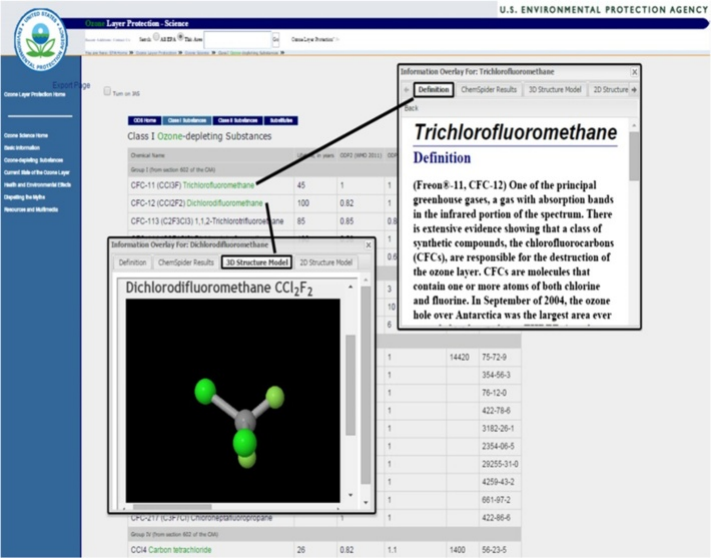
Screen capture of US EPA page for ozone depleting substances after submission to the WHG. Two portlets have been activated and being chemicals, these portlets have 4 tabs, providing different types of information. The top portlet displays the default definition tab, while the lower one shows the 3D structure that contains additional information extracted from the ChemEd DL Models 360 database

Currently, the most common method to acquire additional information when reading a document is to access a search engine and browse the web. This process may be repeated multiple times and becomes time consuming and distracting. Novice readers will often give up and surf to other documents that they find easier to read. The WHG avoids the necessity of leaving the document by allowing readers to embed HG tagged terms in the document, which are the links for displaying content in the WHG Portlet. The WHG Portlet is a JavaScript generated overlay that is superimposed on the document. It is a portal to additional knowledge about the selected item. The reader can activate more than one portlet and move them around the screen (Fig. 2). The portlet may contain tabs that allow the reader to access different types of information associated with the HG tagged term. Some of this information is extracted from specialized databases that are not directly accessed by common search engines, such as chemical structures in ChemEd DL Models 360 [40]. This is where open standards are important, as they allow words (such as chemical names) to be associated with identifiers that enable automatic queries of multiple databases.

**Fig. 3:**
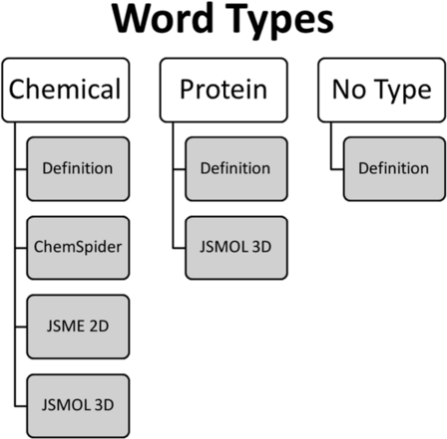
Words in the WHG glossaries are assigned to type categories, with the current types being *chemical*, *protein*, and *no type*. Word types are also associated with a semantic identifier, which allows them to be connected to the content of external databases. The information presented in the WHG Portlet is governed by the word type

The extensible nature of the WikiHyperGlossary architecture allows for the classification of words into types by associating them with semantic type identifiers. Currently, there are three types: “*no type*”, “*chemical*”, and “*protein*”, (see also, Additional file 1, a video that describes these aspects in the context of bulk uploading existing glossaries). All word types have a WHG database identifier, while *protein* and *chemical* word types are also associated with a semantic identifier, which allows them to be connected to the content of external databases. In the case of chemicals, this is the InChI identifier, which also contains additional structural information that can also be used by software agents [41]. The content that is returned to the portlet depends on the glossary that is chosen as well as the type of term, see Figs. 2 and 3. Current types of content include multimedia enhanced definitions, ChemSpider query results, 3D molecular structures and 2D editable structures. The 2D editor tab can bring forth additional tabs containing ChemSpider results for molecules created with the editor. The tabs are described next.

### Definition tab

This is the default tab and contains the original definition stored in the WHG database associated with that glossary. Each definition may have up to 5 different definition text fields, which can contain multimedia content that are either stored in the WHG database or linked externally. Individual fields may be locked or unlocked for editing, the latter providing wiki (user editing) functionality through the Tiny-MCI WYSIWGY editor. Previous versions are stored after each edit, providing a history of each definition. Each definition also contains the option of providing a glossary-wide source citation, which would be used when external glossaries are bulk-uploaded (see glossary management section). A common glossary architecture is to bulk upload an established (canonical) glossary, lock it, and then associate an editable (wiki) field with it (see background information on coupling social to canonical definitions).

### ChemSpider searches tab

Word type *chemical* has a ChemSpider tab, connecting the term (a chemical) to additional information through ChemSpider, which is just one of the ways the WHG uses ChemSpider. When an item of type *chemical* is selected, the item is used to perform a simple search of ChemSpider, which tries to return a list of ChemSpider identifiers. The ChemSpider identifiers are then passed to the GetCompoundThumbnail service to query for thumbnail images of the compounds. Each thumbnail is returned as a 64 bit string which must be decoded. The Perl module MIME::Base64::Perl decodes the string into a PNG format graphics file that is saved to the WHG server. The image is then displayed in the portlet, and becomes a link to the ChemSpider web page where additional information on the compound can be found.

### 3D structures tab

**Fig. 4:**
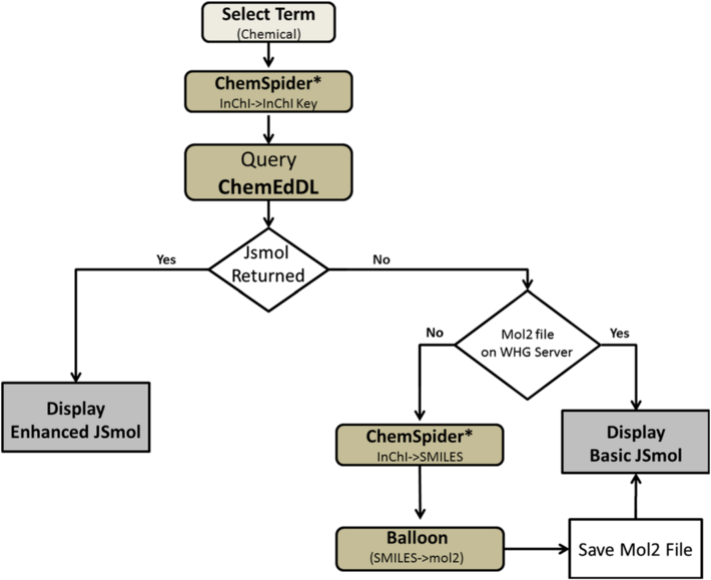
A flowchart depicting the process of presenting a 3D molecule in the JSmol applet when a term with a qualifying ID is selected. ChemEd DL Models 360 database is initially queried and if the chemical is found, the enhanced JSmol is displayed. In the event that the information requested for a chemical term is not found, the WHG has the ability to generate its own structure file from the chemical ID. Open Babel (run internally at ChemSpider) and Balloon, open source chemistry resources, are used to create a mol2 file on the fly. All generated mol2 files are saved on the WHG server to avoid having to create the same file more than once

Terms that are in glossaries and are either of type *chemical* or *protein* have unique identifiers assigned to them. If a type *chemical* term is selected and the 3D tab is clicked, its InChI is queried from the database. This is converted to an InChIKey, a 25-character hash of an InChI geared toward automated operations, which is used to query the Models 360 database of ChemEdDL [41]. ChemEdDL in turn tries to return an enhanced JSmol representation for 3D display in the JSmol software. If a JSmol representation is not available at ChemEdDL the system can generate one dynamically. To do this it first converts the InChI to a SMILES string using ChemSpider’s convert web service which internally uses OpenBabel [42]. The SMILES string is then sent to Balloon [43] which creates a mol2 file with the 3D coordinates. The mol2 file is saved so that it only needs to be created once. The location of the file is then sent to the JSmol application for display. This process is depicted in Fig. 4.

If the word type is *protein* then the system retrieves the Protein Data Bank (PDB) id for the protein that is selected and the PDB id retrieves the PDB file from the RCSB website [44]. This file is submitted to the JSmol application to render the 3D structure of the selected protein.

### 2D structures tab

**Fig. 5:**
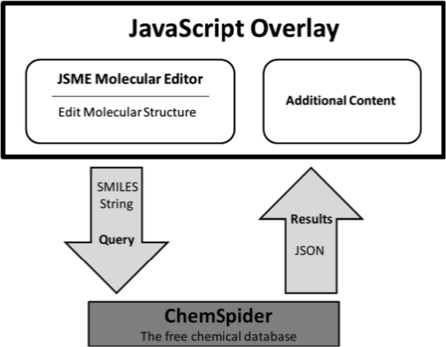
Overview of the process of querying ChemSpider using the JSME editor. In the 2D structure tab of the WHG Portlet the user can alter the 2D structure of the selected molecule. A link at the bottom of the WHG Portlet grabs the resulting SMILES string using JSME’s API. The SMILES is converted to an InChIKey which it uses to query ChemSpider. If the resulting string is an actual chemical in the database, the query results are shown. Otherwise a message saying “no results” is shown

Word type *chemical* has a 2-D structure tab that launches the free JSME molecular editor [45]. To load the 2D chemical structure the associated InChI is sent to CACTUS (CADD Group Chemoinformatics Tools User Services) [46], which provides a service that converts the InChI strings to JME format in order to load molecular structures into the JSME viewer. Once the 2-D structure of the molecule is loaded, JSME also allows the molecule to be edited into a new chemical. A clickable link at the bottom of the window submits the SMILES string for the new chemical to ChemSpider. JavaScript code was written to use the JSME API (getSMILESs()) to grab the SMILES string of the current chemical structure in the applet. The string is sent back to the server where it is converted to an InChI string and an InChIKey using ChemSpider’s web service which internally uses Open Babel. The InChIKey is then used to query ChemSpider’s database and have it return a PNG thumbnail of the compound if it exists in the database. The thumbnail is linked back to ChemSpider with additional information on the newly created structure. The information is presented in a new tab (Fig. 5).

### Content management

The content management system is broken into two components, User Administration and Glossary Management.

### User administration

The user management portion of the system supports adding, removing, and updating privilege levels of users, including those with administrative authorization. Different roles permit different levels of access to the WHG Database. The basic guest level allows processing documents with any available glossaries through the web portal and does not require an account, however additional privileges require account authorization. Typical profiles are “authorized user” for adding/editing definitions and uploading multimedia (to contribute to the wiki) and “administrator”, for adding users and creating glossaries, including the bulk upload of existing glossaries.

### Glossary management

**Fig. 6:**
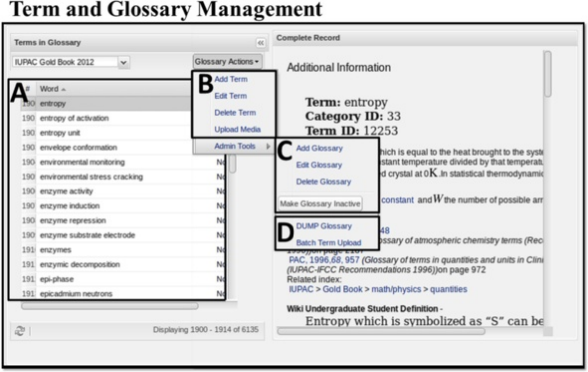
The WHG term and glossary management interface allows authorized users to edit, add, and delete terms and glossaries. **a** A panel displays all the terms in a selected glossary. **b** When a term is selected the user then has several options. **c** Functionality that requires a user to have another level of access rights includes full glossary adding, editing, deletion or inactivation. **d** Full glossaries can be dumped to a text file or bulk imported if in the correct XML format

Administrators can create glossaries. Once logged into the system a link to the glossary management panel becomes available (Fig. 6). Section A of Fig. 6 shows an alphabetical list of terms in the IUPAC Gold Book 2012 glossary that also indicates the word type (*No Type*, *Chemical & Protein*) for each entry. Authorized Users (contributors to the wiki) have access to the features in section B, allowing them to add, edit and delete terms, and to upload multimedia files. See Additional file 2 for a video on how to upload a definition from a MS Word document, and Additional file 3 for a video on how to upload an image. Section C in the “Admin Tools” allows for the administration of glossaries. Administrators can set the number of fields available to a term, if the field is editable (a wiki definition) or locked (a canonical definition), and if there is a source citation for all canonical definitions associated with the first field of the glossary. An additional level of permissions allows for the downloading of an entire glossary as a csv file, and for the bulk uploading of external glossaries as XML files.

The Ext JavaScript library is used extensively to implement this component. Information from the database to populate the forms is done through the use of AJAX, which is relatively quick and responsive. The information is converted to JSON format by the server and sent to the browser.

### Batch term upload

**Fig. 7:**
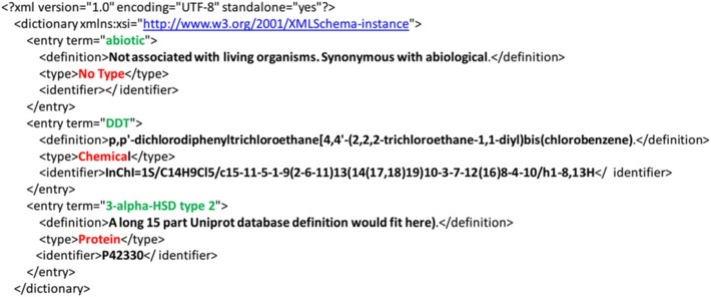
Example partial glossary in the required XML format for bulk uploading to the WHG. The first word is a normal definition, the second a chemical, and the third a protein. Definition 1, 2, and 3 are the actual definitions of the words from the glossary being uploaded

A powerful feature of the WHG is the ability to upload existing glossaries, associate a citation with all definitions and lock them so they cannot be edited, while also providing the option of associating up to four editable wiki-fields with each locked definition. A bulk upload feature allows an entire glossary to be uploaded as an XML file. This requires preprocessing existing glossaries, which can be obtained as documents in a variety of formats and file types (see Additional file 4). The task is further complicated by the need to identify the word type of a glossary term, and obtain its semantic identifier prior to generating the uploaded XML file. Figure 7 shows the extensible XML schema for a glossary definition.

### Preprocessing bulk glossaries

**Fig. 8:**
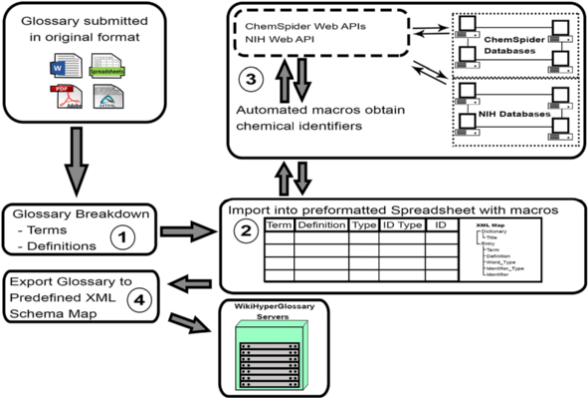
Four step process for preparing a glossary for bulk upload to the WHG. **1)** Take the original glossary, which can come in a variety of formats. **2)** Map the terms and definitions to the columns of a macro-enabled Excel. **3)** If the glossary has chemicals, one needs to identify which words are chemicals, and assign their InChI. Shown is how chemical InChI API services (ChemSpider and NIH) are utilized to automate the process. **4)** The final step is to export an XML file that can be bulk uploaded to the WHG. The Bulk Glossary Upload supporting document describes these in detail for a glossary containing chemical terms

Central to the strategy of improving reading comprehension by coupling social definitions to non-editable canonical ones is the ability to easily upload existing glossaries to the WHG, and then enabling wiki-definitions to be associated with them. This allows for the extension of the WHG to glossaries of different disciplines and makes the WHG a true interdisciplinary information literacy technology. There are two major challenges here. First, there is no standard format or document type for existing glossaries, necessitating an adaptable preprocessing workflow. Second, “word types” need to be identified and semantic identifiers assigned for appropriate words. Right now there are only two word types, chemicals and proteins, but this feature is extensible to other disciplines. Figure 8 shows an adaptable workflow for this process, using the identification of the InChI semantic identifier for the word type “chemical” as an exemplar. The objective of this process is to generate an XML file with a schema containing the glossary information that can be uploaded over the web to the WHG, and the video in Additional file 1 describes this process in detail.

Figure 8 shows the four step glossary preprocessing workflow that is described in detail in the document of Additional file 4. The first step is to take the original glossary, which can come in a variety of formats, and map the terms and definitions to the columns of a macro-enabled Excel Spreadsheet (Additional file 5). If the glossary has chemicals, one needs to identify which words are chemicals, and assign their InChI. Step 3 shows how web API services do this and further details are available in Additional file 1. By running parallel processes using ChemSpider and NIH APIs one can compare results to gain a greater degree of confidence in the assignments. If there are other word types, a new protocol would need to be developed to take advantage of resources of that discipline to assign the appropriate semantic identifiers. The final step is to export an XML file that can be bulk uploaded to the WHG.

## Results and discussion

**Fig. 9:**
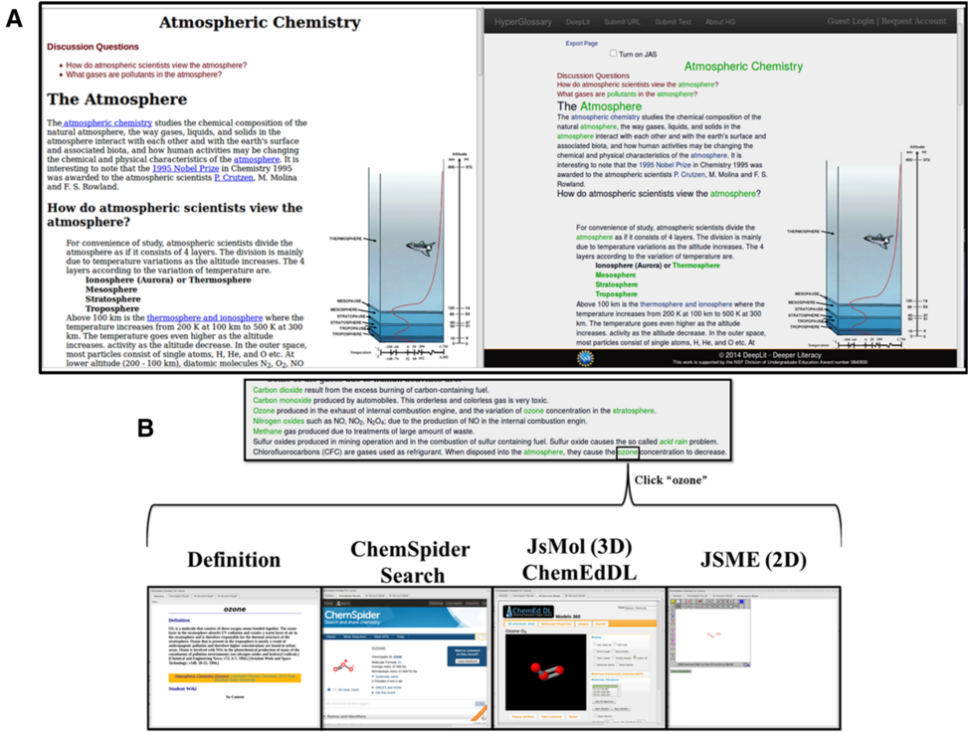
Overview of the process of querying ChemSpider using the JSME editor. **a** Shows a page pre- and post-processing. Terms in green where found to be in the selected glossary. **b** These terms can be selected and content pulled from the server are bought to the WHG Portlet. In this case the term “ozone” was selected and the WHG portlet appeared with four different tabs which include a definition of the term, a ChemSpider search was performed, a 3D model was generated, and in the final tab a molecular editor with the 2D structures was generated

The WHG allows any user the ability to submit a web URL or text to be processed. Figure 9 shows a webpage before and after processing. Upon the selection of an HG tagged term (green) a JavaScript overlay, the WHG Portlet, pops up with a definition, and depending on the type of word, tabs to further information. In Fig. 9 the term “**ozone**” was selected, which being a chemical contains four tabs that are displayed in the figure. The first tab is a definition, the second the results of a ChemSpider search, the third a 3D structure displayed by the JSmol application retrieved from ChemEdDL, and the fourth tab a 2D structure displayed in the JSME application. The JSME tab can create more tabs with ChemSpider searches of molecules in the editor.

### Enhancing literacy: coupling social definitions to canonical definitions

Can the WikiHyperGlossary enhance literacy in the Google Age of instant access to information, including expert-level documents in a novice’s distal knowledge space? The WHG architecture supports a strategy that connects expert level documents to novice level background information by inserting hyperlinks within documents. Can this be done at a sufficient density to provide the implicit knowledge that the expert authors assumed the reader possessed? The strategy is to parse a document through a glossary of the document’s knowledge domain, effectively using the lexicon of the domain to connect the document to resources of the domain. The system then couples multimedia social (wiki) generated novice-level definitions to expert-level canonical definitions generated by learned societies of the domain. The objective is not just to provide the definition of a word (explicit knowledge). But to create enough hyperlinks in the document providing novice-level content coupled to expert level definitions, so the novice acquires the background (implicit knowledge) that enables comprehension of the expert-level document [47]. See the video of the Additional file 6.

**Fig. 10:**
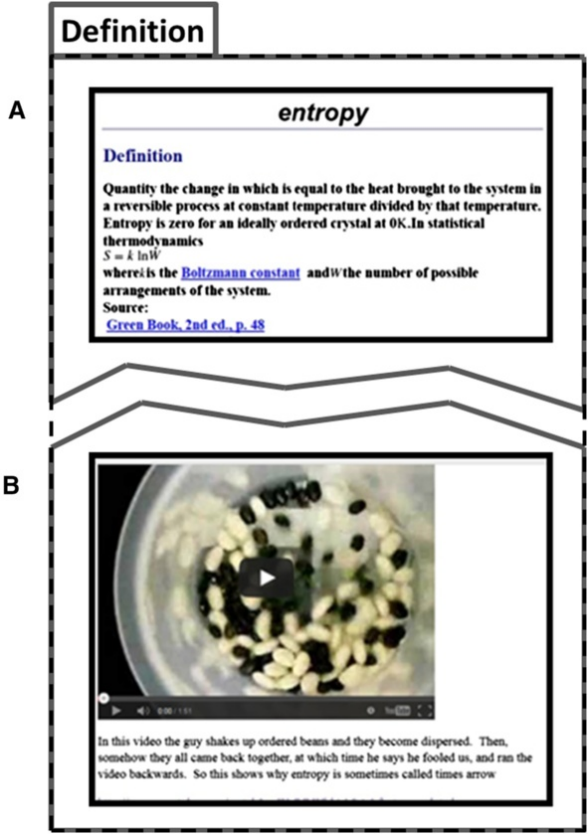
Screen shots of the WHG IUPAC glossary definition for entropy. On top **a** is the canonical definition that is displayed when viewed in the WHG Portlet. As the novice scrolls down they reach part **b**, which has an imbedded video that was created by a freshman student at the University of Arkansas at Little Rock. These screen shots are described in the video of Additional file 6

For example, a novice reading an article on thermodynamics might not understand words like entropy, enthalpy, etc., and fail to benefit from the article. After running the document through an appropriate glossary, like IUPAC’s Gold book, the novice would have instant access to expert-level canonical definitions, but being expert level, these alone could cause even more confusion. Using entropy as an exemplar (see Fig. 10), the novice finds two definitions in IUPAC’s Gold book definition (top of Figure) based on Clausius’s (*S* = q_rev_/T_abs_) and statistical thermodynamics, *s* = klnW. Neither of these are designed to fulfill the information needs of the novice (these are expert-level definitions). Below these the WikiHyperGlossary embeds a social-generated definition with embedded videos targeting background knowledge at the novice level. After reading sufficient multimedia wiki-definitions scattered throughout the document the novice acquires the missing implicit knowledge and has enhanced understanding of the document.

### Knowledge discovery in a molecular editor enabled semantic framework

There is a fifth type of tab in the WHG Portlet that can be activated with the JSME 2D editor, which populates the portlet with the ChemSpider search results for whatever molecule was in the editor when it was activated. A user of the WHG can add as many of these new tabs to the portlet as they desire. From an education perspective this could potentially be classified as a type of semantic web interface capable of inductive reasoning based discovery activities that could be used in classrooms. Many semantic web applications utilize RDF triples and OWL based activities, which model deductive reasoning in the sense that knowledge is abstracted through pre-existing formalizations embedded into the online content. The question arises, does the semantic web support knowledge generation through inductive reasoning processes where the knowledge framework evolves out of exploratory based behavior of the novice-learner? We believe through the use of chemical identifiers, open access databases and open source molecular editors the WHG extends this capability to digital documents and web pages that contain chemical entities, in the form of inductive reasoning processes generated through a semantic discovery framework.

**Fig. 11:**
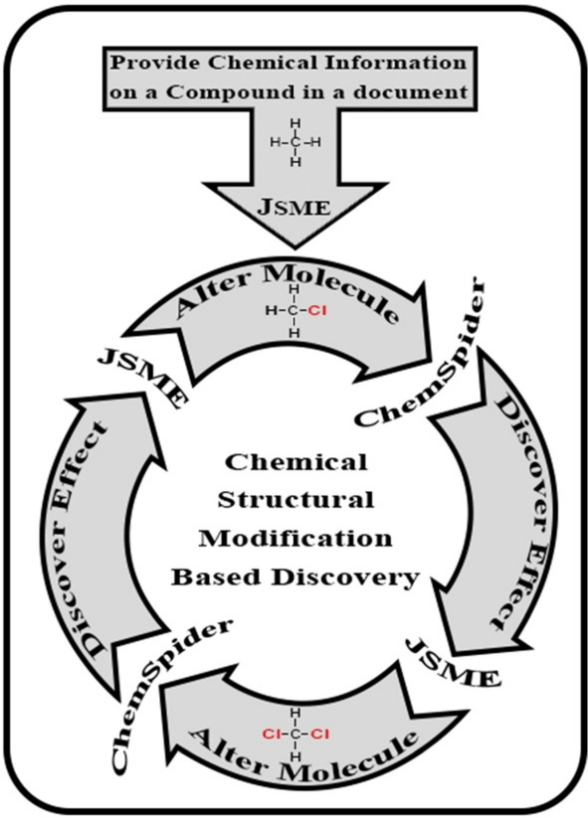
Structural modification based discovery process where readers can question a statement concerning a molecule in an article, get published data on the molecule, change the molecule, and get data on the new molecule, all without ever leaving the article. See Additional file 7 for a video demonstrating this process

A person reading an article which describes a reaction involving methane could ask how does successively chlorinating the hydrogens affect the boiling point? The WHG provides the information through using the JSME molecular editor to query the ChemSpider search services, where the student can change a hydrogen to a chlorine and successively repeat the process (Fig. 11). Each time the molecule is modified and searched, a new tab appears with the results of the new search. While reading an article a student could quickly convert the methane to CH_3_Cl, CH_2_Cl_2_, CHCl_3_ and CCl_4_, and have 5 tabs, one for methane and one for each of the modifications. This could easily be extended to other properties, and without ever leaving an article, answers to questions like these can be discovered, and general principles could be developed in an inductive fashion. See Additional file 7 for a video demonstrating this process.

### Integration into Jikitou

Although the WHG is a standalone application designed to process documents, the functionality of the WHG can be integrated into other software applications. The WHG server’s ability to pull information from multiple resources can be used to enhance other systems. To that end the WHG has been successfully integrated into Jikitou (www.jikitou.com), a biomedical question answering system [37]. In this era of large scale processing of Next Generation Sequencing, which includes RNA-Seq and Whole Exome Sequencing, and a multitude of other molecular profiling modalities, biomedical researchers are often left with a set of genes that show signs of biological significance. The next step is often to determine what these genes' likely roles are, and how they may be impacting the disease or condition of interest. Initially, that investigation starts with a thorough search of the published scientific literature. Jikitou is a tool for biomedical researchers, which supports that initial information search.

Researchers are often interested in how the scientific literature supports and elucidates potential links between key molecules of different molecular modalities such as proteins, and genes to find insightful connections with a diseases or condition. Jikitou takes a user’s query posed in the form of a natural language question and returns a list of potential answers from sentences taken from biomedical abstracts. The corpus that is used as the pool of potential answers contains sentences that have at least two biomolecules and an interaction indicating term. Jikitou uses natural language parsing to build a query that returns relevant answers without requiring the users to build a cryptic query string of keywords. Users of Jikitou can choose different glossaries that will identify terms that can be linked to additional information in potential answers. Just as in the WHG, the user can click on highlighted words to activate a WHG Portlet to additional supportive information.

**Fig. 12:**
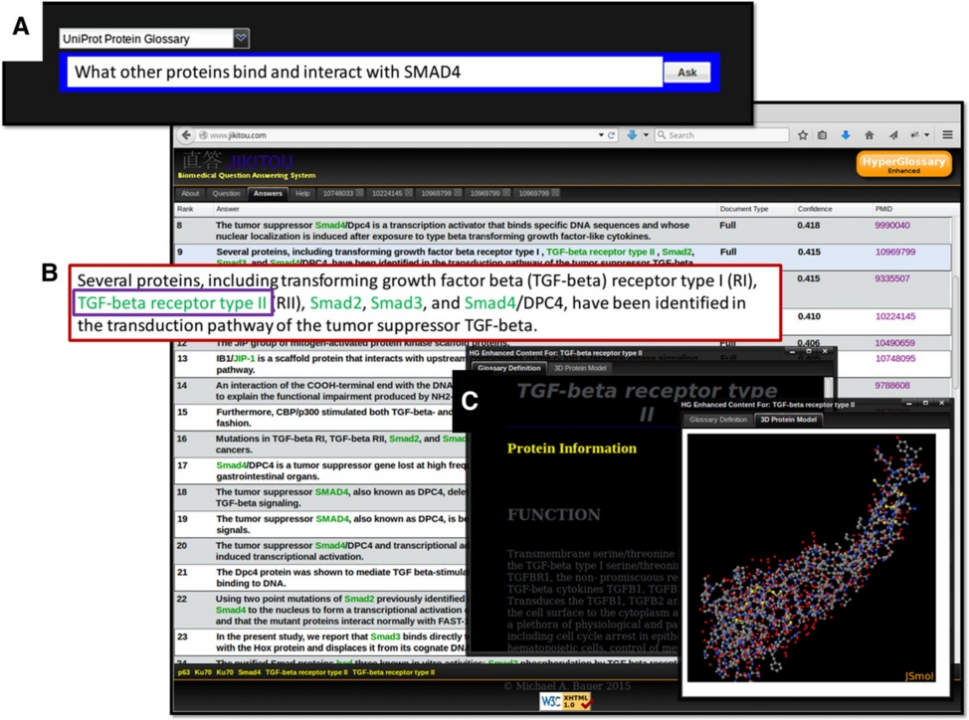
The WHG functionality has been successfully integrated into the Jikitou Biomedical Question and Answering System. This application brings back potential answers to questions asked in natural language to biomedical questions. These answers are enhanced with the WHG Portlet which brings back additional information on terms in the answer that are found in the chosen glossary. **a** A question is asked to the system and the UniProt glossary is selected. **b** A set of potential answers are returned and proteins that were matched in the glossary in the answer text are identified by a change in font color to green. In this example the protein TGF-beta receptor type II was selected. **c** The WHG Portlet appears with two tabs. The first being a functional description of the protein and the second a JSmol applet with the protein structure loaded

Figure 12 demonstrates an example of using Jikitou. A question is asked to the system and the UniProt glossary is selected. Here the question asked is “What other proteins bind and interact with SMAD4”. Once the question is submitted a set of potential answers are returned and protein names that were matched in the glossary to those found in the list of potential answers are identified by a change in font color to green. In this example the protein “TGF-beta receptor type II” was selected. The WHG Portlet appears with two tabs. The first being a functional description of the protein and the second a JSmol applet with the protein structure loaded. This ability to quickly get a functional description and structure of a particular protein or gene into the current window of results without requiring additional queries to outside resources has the potential to increase the efficiency of the literature search, and greatly increases the usefulness of the Jikitou system.

## Conclusions

The late twentieth century corpus of scientific and cultural knowledge predominantly existed in the form of the printed text. Early twenty-first century digital technologies created new literacy challenges. Some deal with reading comprehension and the ease of obtaining printed documents in one’s distal knowledge space. Others deal with new database enabled forms of information management, manipulation and communication. Information literacy technologies are evolving to tackle new literacy issues and opportunities. The WikiHyperGlossary is a digital information literacy technology that has been developed to assist humans in understanding printed documents in the chemical sciences by embedding dynamic hyperlinks that connect them to new resources of the evolving world of digital content.

The WikiHyperGlossary (WHG) enhances reading comprehension by using the lexicon of a discipline to generate dynamic links in a document to both canonical definitions of learned societies and social generated multimedia definitions that can provide implicit information the document did not explicitly provide. By associating semantic identifiers like the InChI with words (chemicals) the WHG can also connect documents to a variety of software agents and databases. Technologies like the WHG also have the potential to enable new forms of virtual cognitive artifacts [48] that can impact human reasoning processes. This is evidenced by the Molecular Editor Enabled Semantic Framework, which could enable knowledge discovery via inductive reasoning processes connected to the printed corpus.

A key concept behind the implementation of the WHG is extensibility, both into other knowledge domains, and into other software agents. The WHG code that this paper describes is available at GitHub and has been successfully integrated into the Jikitou Biomedical Question and Answering System. The work presented in this paper is essentially proof-of-concept work, and to truly impact 21^st^ century literacy issues, technologies like the WHG need to be extended into other knowledge domains and integrated into knowledge acquisition workflows, like internet search services.

A fundamental niche that an information literacy technology like the WHG fits lies with connecting the knowledge stored in the printed corpus of the past to the future knowledge of the evolving digital corpus. A technology startup, DeepLit, is evolving out of this work. DeepLit stands for “Deeper Literacy: Connecting Documents to Data and Discourse”. [49] DeepLit’s mission is to move WHG technologies into the public sector of information acquisition and assist the public with 21^st^ century literacy challenges. Anyone who is interested in contributing to, or using this technology, should contact the corresponding author, Bob Belford.

## Availability and requirements

**Project Name**: WikiHyperGlossary

**Project home page**: www.hyperglossary.org

**Also available at**: whg.chemeddl.org

If you would like to contribute or run on your own server we have the following options:

- An Amazon instance image, running Ubuntu 10.04, which has been made public with the following name and id:

***AMI ID****: ami-822bf7eb*

***AMI Name****: WHG*

- **GitHub**: https://github.com/DeepLit/WHG

**Programming Language:** Perl, JavaScript

**License**: Apache Version 2.0

**Any restrictions to use by non-academics**: None

## Additional files

Additional file 1:
**Video on how to Bulk Upload a glossary.** This video also shows the structure of the XML file and different form fields of the definition that maps to the corresponding XMLtags. Also available at http://youtu.be/ptX9tIrqcEE. Additional file 2:
**Video demonstrating how to post a wiki-definition to the WikiHyperGlossary that was created in a MS Word Document.** Also available at http://youtu.be/ckDSHMNd-u4. Additional file 3:
**Video demonstrating how to upload an image to a definition.** Also available at https://www.youtube.com/watch?v=I5xEj-OpUCQ. Additional file 4:
**Description of the Glossary Preprocessing workflow.** This file describes the steps of Fig. 8 in detail, including the use of ChemSpider and NIH APIs to identify if a term is a chemical, and if so, to obtain its InChI. Additional file 5:
**Macro enabled XML generating spreadsheet (item 2 of Fig.** **8** **)**. Additional file 6:
**Video on how to improve reading comprehension by coupling social definitions to canonical definitions.** Also available at http://youtu.be/KrSMuzLYycs. Additional file 7:
**Video of Molecular Editor Enabled Semantic Framework.** Also available at http://youtu.be/e3Y93Im9WKo. Note, this video uses the Java based JChemPaint applet, which has been changed to JavaScript JSME, and also shows the JavaScript Automated Search functionality that is described in the background section of this article.

## Abbreviation

WHG: WikiHyperGlossary
