# Supplementary material for: WikiHyperGlossary (WHG): an information literacy technology for chemistry documents
Source: J Cheminform. 2015 May 22;7:22. doi: 10.1186/s13321-015-0073-7 (PMC4477724; doi:10.1186/s13321-015-0073-7)
Supplement: Additional file 4: — Description of the Glossary Preprocessing workflow. This file describes the steps of Fig. 8 in detail, including the use of ChemSpider and NIH APIs to identify if a term is a chemical, and if so, to obtain its InChI. [file 13321_2015_73_MOESM4_ESM.docx]

**A framework demonstrating the process of converting a convential glossary to a WHG uploadbale glossary and associating semantic identifiers to terms.**

**Overview:** This document describes the workflow for taking an existing glossary and uploading it to the WikiHyperGlossary (WHG) database with attached chemical identifiers. A list from the IUPAC Glossary of Terms in Immunotoxicology Annex II[1] is used to exemplify this process. Additional steps required for identifying which words are chemicals and associating InChI identifiers is explained in several steps throughout this document. Figure 1 outlines the four basic steps to this workflow. It should be noted that this process can be extended to new word types if there are semantic identifiers associated with them, including catalog databases. If the glossary is just terms and definitions, step 3 is skipped.

**
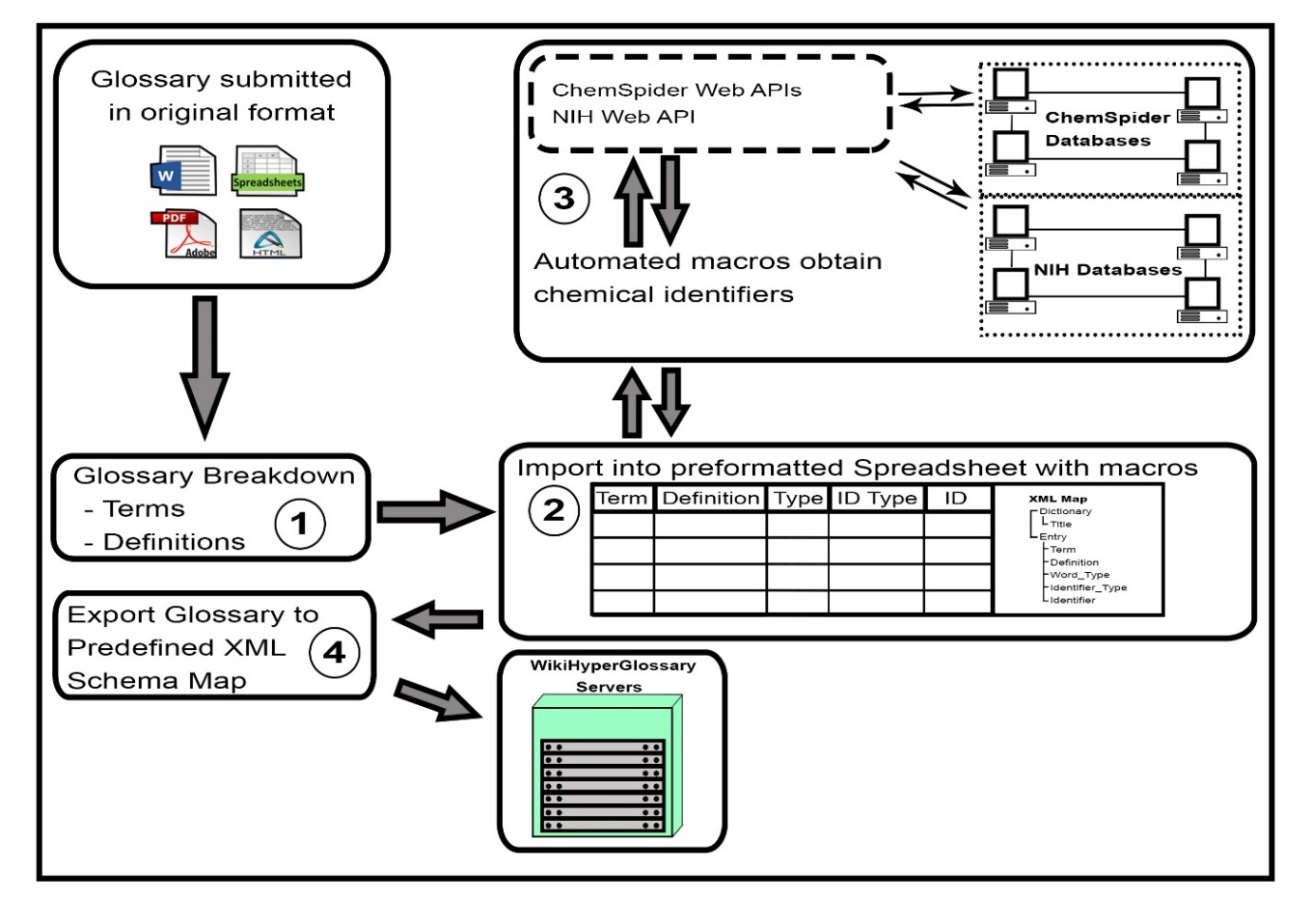
**

Figure 1: Workflow for preprocessing glossaries for bulk upload.

**Steps for Converting PDF of IUPAC Immunotoxicology Glossary Terms to XML with InChIs**

[1. Glossary Breakdown](#Glossary_Breakdown)…………………………………………………………………………………………………………………… p. 02
 Glossaries come in a variety of formats and need to be separated by terms and definitions.

[2. Import into Preformatted Spreadsheet with Macros](#Import_into_Preformatted_Spreadsheet)…………………………………………………………………….p. 03
Map Glossary Terms and Definitions to appropriate columns of attached *Chemical Glossary Builder* spreadsheet (additional file 5).

[3. Automated Macros Obtain Chemical Identifiers](#Automated_Macros_Obtain_Identifiers)……………………………………………………………………………p. 4-7
 Identify which words are chemicals, and acquire their InChI identifiers.
 Other word types can also achieve the same goal with a different type of semantic identifier

[4. Export Glossary to uploadable XML file](#Export_Glossary_to_Predefined_XML_Schema)………………………………………………………………………………………..p. 8
 Use embedded function to map columns to XSD schema and generate XML file.
 Upload XML file over the web to the WikiHyperGlossary database.

**1. Glossary Breakdown**

The first step is to separate the glossary content into terms and definitions that can then be mapped to the columns of a spreadsheet in step 2. The difficulty with breaking down glossaries is that they are often submitted in different formats and file types. The most common file types are pdf, doc, xml, txt and html. The easiest to convert are the spreadsheet file types, as they only require a copy and paste command to transfer a glossary into the preformatted spreadsheet. Word processing documents, web pages and pdf files require more steps that are often unique to a particular glossary, because even documents of the same file type can have a wide variety of formats.

To breakdown glossaries that are saved in these non-spreadsheet formats the process begins with opening the file in the software program that it was designed with. From this point the glossary can often be exported or saved as a Microsoft Word Document if this was not the original format. Advanced find and replace strings are then used in Microsoft Word to analyze key differences in the formatting of the text to select either terms or definitions. The strings used to search are often a combination of headers, paragraph marks, font styles and line breaks. Once the individual glossary components are selected, a copy and paste command transfers the glossary to our spreadsheet. Figure 2 demonstrates a simple process to apply this separation when selecting a component.


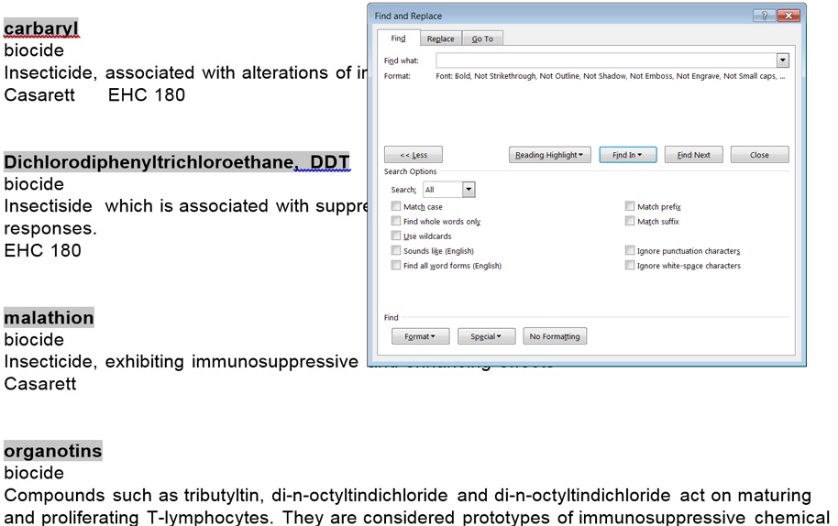


Figure 2: Microsoft Word’s Find and Replace feature is used to highlight an entire component of a glossary based on formatting. In this example a specific search criteria is used to select all words that are bold in the glossary. Because this glossary used bold formatting to represent the terms, it is used as a way to convert the glossary into a spreadsheet for further processing. Then a search is performed for words that are not bold to allow for the importing of the respective definitions to these terms that is put into a separate column of the same spreadsheet.

**2. Import into Preformatted Spreadsheet with Macros**

In the previous step the glossary was broken down into terms and definitions and these now need to be mapped to the “*Chemical Glossary Builder*” (additional file 5) spreadsheet that is attached to the article as a supporting Document. This spreadsheet contains several macros to automate some of the steps involved with preprocessing a glossary. Macros allow us to perform a large number of computer commands within a short amount of time.

This step starts with the completion of transferring the terms and definitions of a glossary from the original format into the *Chemical Glossary Builder* preformatted spreadsheet that has columns mapped using a WHG specific XML Schema. Glossary terms are placed into column 1 and their respective definitions are placed into column 2 and this is shown in figure 3. It is also necessary to check that all of the definitions and terms are placed onto the same row, or the terms will have the wrong definitions in the final XML file. When all of the pieces are put into place the user must then make sure the developer tab is enabled in the Microsoft Excel Options. This will allow the user to enable the macros needed in step 3, along with the ability to export to XML in step 4. Each version of Microsoft Excel is a little different and a quick search on the internet for that version on how to enable macros and the developer tab will give a step by step set of directions.


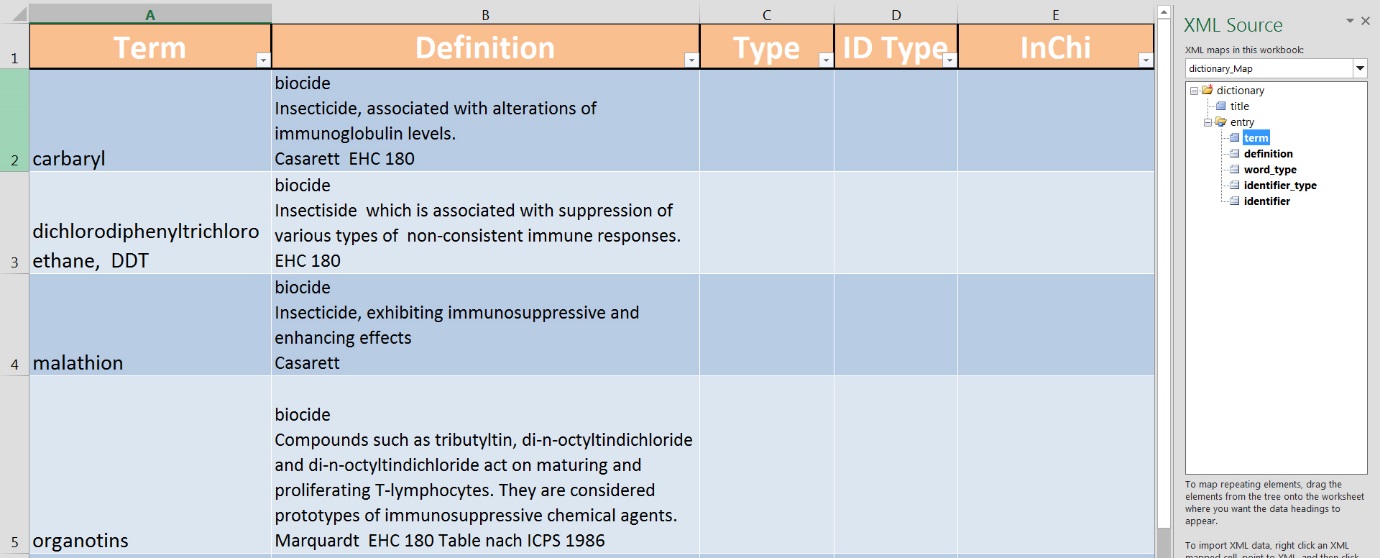


Figure 3: This is an example of what the glossary will look like once it is imported into our preformatted spreadsheet for further preprocessing. The terms will be separated from the definition and one another through the use of columns and rows. This spreadsheet is pre-mapped for easy exportation to an XML format as shown with the glossary structure on the right side of the image.

**3. Automated Macros Identify Chemicals and Obtain Chemical Identifiers**

One of the ways that the WikiHyperGlossary communicates with databases and software agents is through the use of an InChI (IUPAC Chemical Identifier) that is associated with chemical terms in a glossary. Therefore, it is important that the identifier for each chemical in a glossary be correctly identified and placed into the final XML format before the bulk upload process. We use Web APIs provided by ChemSpider and NIH to retrieve these identifiers.

1. The first step of retrieving identifiers is to call up a macro function that performs a Web API search with ChemSpider to retrieve the CSID (ChemSpider ID). To get this result, the cells in the column with terms is used as a search string along with inserting an alphanumeric token to verify access permissions. This token is given to registered users of ChemSpider for access to use these web API services. Figure 4 demonstrates this step by showing that the inputs can be retrieved similar to any other function in Microsoft Excel. The code for the function that defines the command is also included.


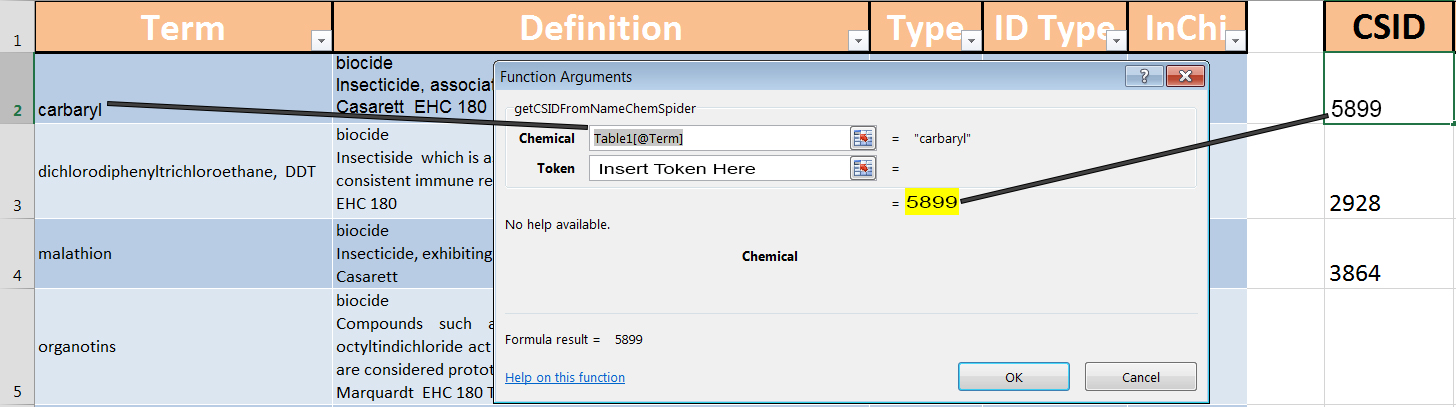

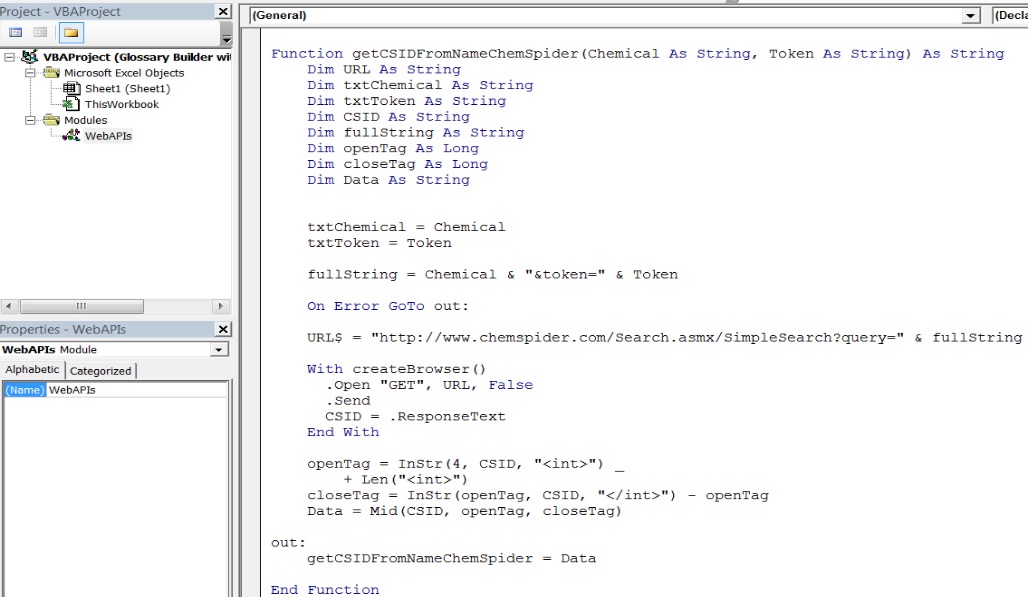


Figure 4: This image shows how a macro function can be called within the spreadsheet to retrieve the ChemSpider ID for a particular chemical name via a Web API provided by ChemSpider. The code that defines this function is also shown at the bottom.

1. After retrieving all of the CSID values for terms that are chemicals, a separate web API of ChemSpider is used to retrieve the InChI. A second macro takes the CSID as the string input and retrieves the InChI as a result. This service also requires a token given to registered users with ChemSpider. The CSID that was used previously to retrieve the InChI is only an intermediate step for the final results and is not included in the XML file created in step 4.

Figure 5 highlights what the function box looks like and where to place the CSID and Token Input. The CSID can be selected by clicking in the field and then selecting the cell with the relevant value to be used as the search input. This function can then be applied to an entire column of a glossary by clicking the bottom right corner of a completed cell and dragging downward.


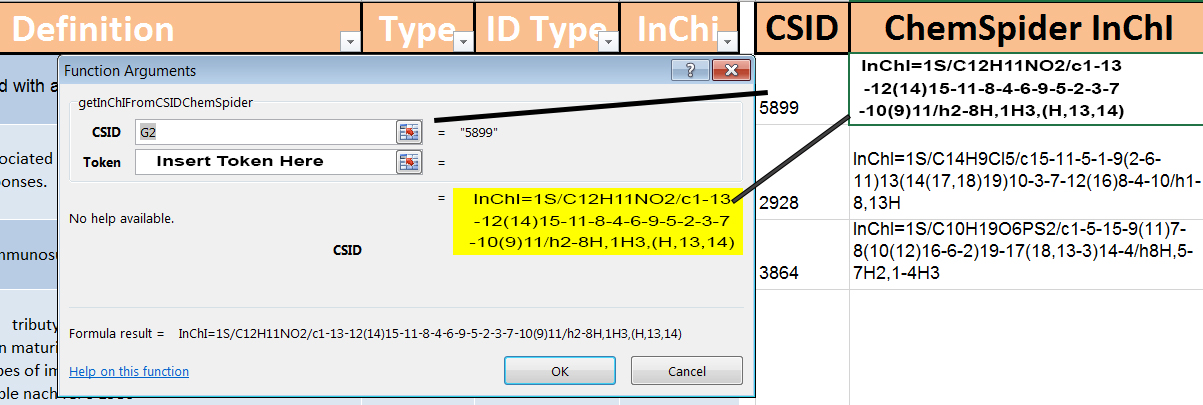

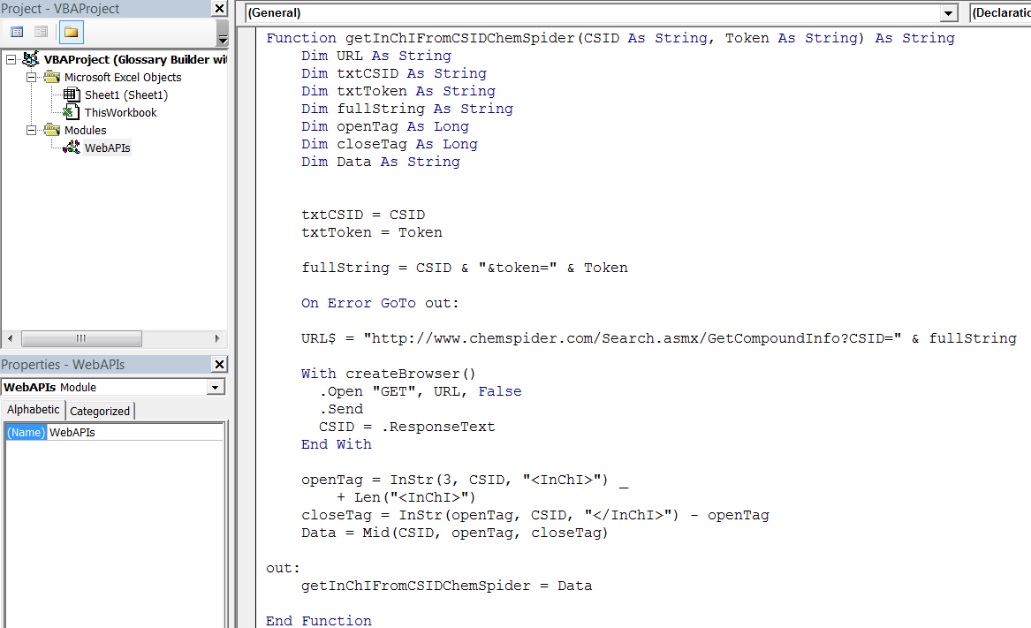


Figure 5: Demonstrates how a macro function can be called within the spreadsheet to retrieve the InChI from the ChemSpider ID via Web API provided by ChemSpider. The visual basic code that defines this function is also shown.

1. The next step is a parallel process for obtaining an InChI that uses a Web API provided by NIH, which is used later to compare with the value from ChemSpider and minimize associating the wrong identifier with a particular chemical term. This service does not require a token and the only input string needed for the function is to select the cell containing the term. Figure 6 shows a representation for how this function works.


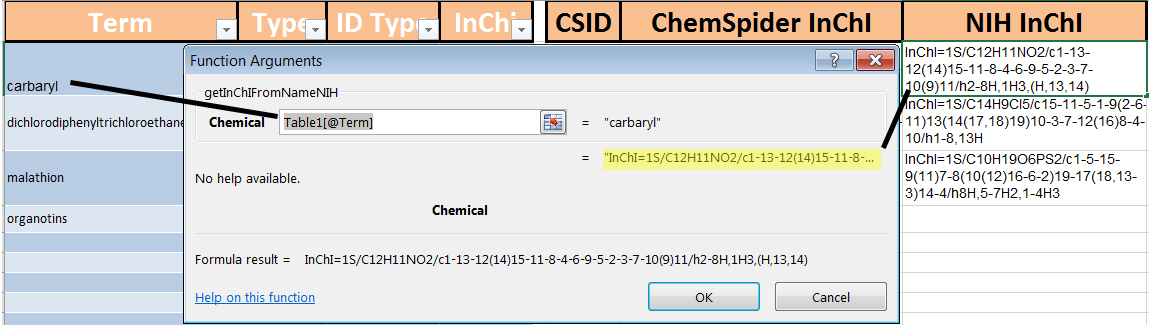


Figure 6: A macro function can be called within the spreadsheet to retrieve the InChI from a chemical name via web API provided by NIH to be used in cross referencing sources for accuracy.

1. The last step involves using a feature in Microsoft Excel that provides conditional formatting to the spreadsheet. This has been set up in a way that will take the InChI sources from both ChemSpider and NIH and compare them to make sure that they are the same. The InChIs that match will turn the font formatting to a light color that is hard to see. The InChIs that do not match will have a black font that is easier to see. This allows for the user to quickly scroll down a long list of identifiers and pick out the ones that need further review to decide which is correct. It also allows for the identification of terms that may have been identified as a chemical by mistake. Figure 7 shows this process below.


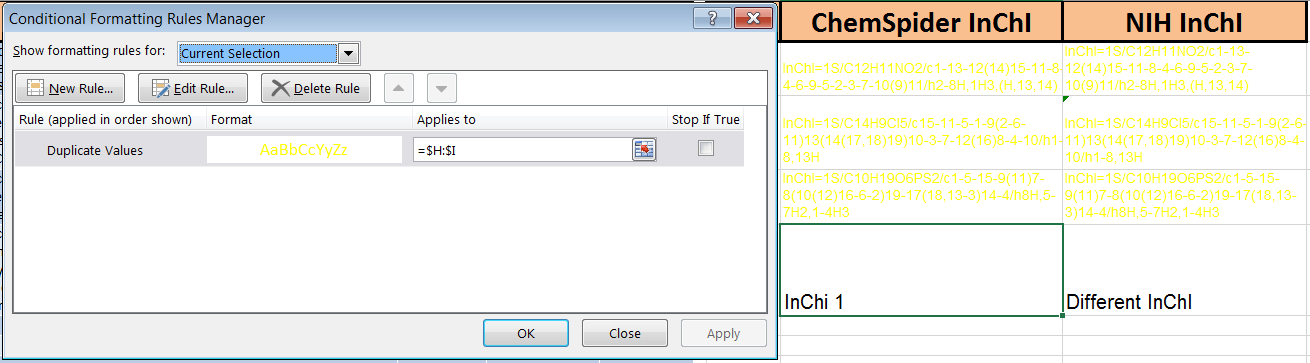


Figure 7: In order to reduce the number of errors with retrieving incorrect chemical identifiers, conditional formatting in Microsoft Excel is used to compare the two different sources. This works by changing the font formatting of the identifiers that match in both sources with those that do not match. The sources that match are brightly colored and the sources that need further checking are left in black. This makes it easier to quickly scroll through a big glossary and find possible errors.

1. After finding all of the identifiers for the chemical terms one can copy and paste the InChIs to the 5^th^ column of the spreadsheet that is mapped to the XML Source Map. Columns C and D of figure 8 will be blank at this point.

The easiest way to fill in these columns is to sort the entire glossary by the InChI column which will put all of the terms with identifiers at the top that have been identified as chemicals. Column C Identifies the word type (chemical), while column D identifies the Identifier type (InChI). This process can be applied to other word types, like “protein”. The terms that are not chemicals or proteins will be sorted at the bottom allowing the user to then put “No Type” into those cells.

When all of the cells are completed the glossary is then put back into the original alphabetical sorting by term. Each column and row at this point is now formatted to match the XML Map to the right as shown in figure 8. This is a representation of what the final spreadsheet should look like.


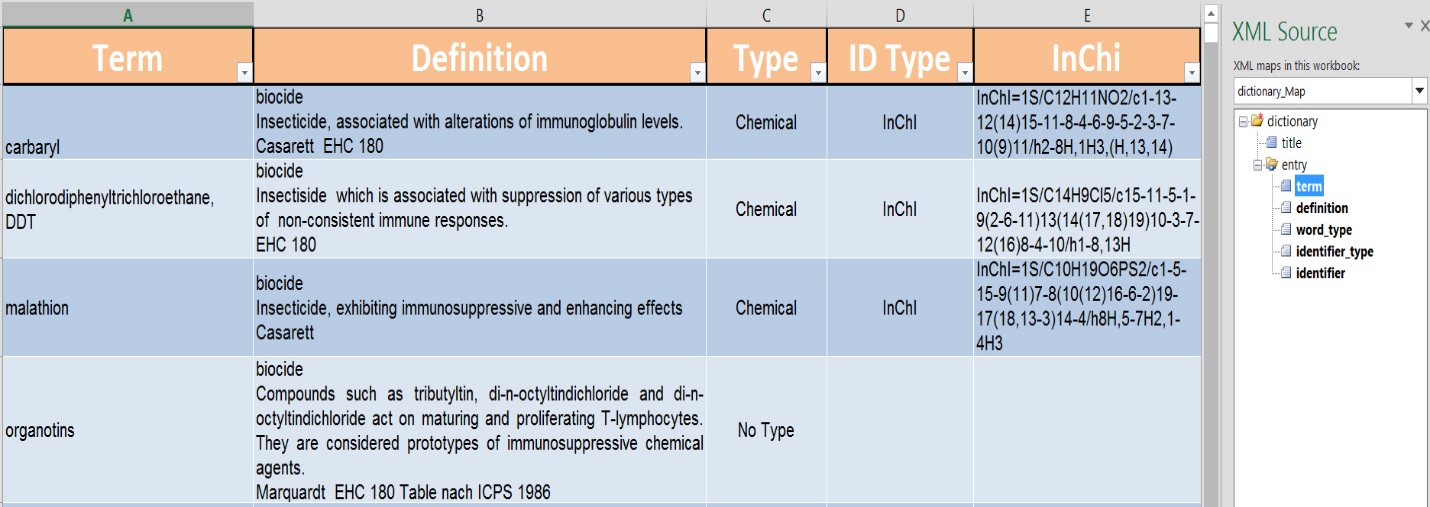


Figure 8: Once all of the identifiers have been retrieved and checked for errors, the additional columns are completed to define the word and identifier types. These columns are mapped to the XML Source and the spreadsheet is saved as a final version that can be edited later if needed. The last step is to export all of the data in the rows and columns to this final XML format explained in step 4.

**4. Export Glossary to Predefined XML Schema Map**

The last step of preprocessing a glossary is to export the glossary from the spreadsheet format to XML. In order to export a glossary, the user can access the developer tab in Microsoft Excel and locate the section for XML. There is a button called “Export” that allows the user to select where the file can be saved, and generate a glossary XML file that can be recognized by the WHG for bulk upload. A sample XML is shown in Figure 8 for the Immunotoxicology List of Chemicals and is uploaded as a supporting document called *Sample XML*.


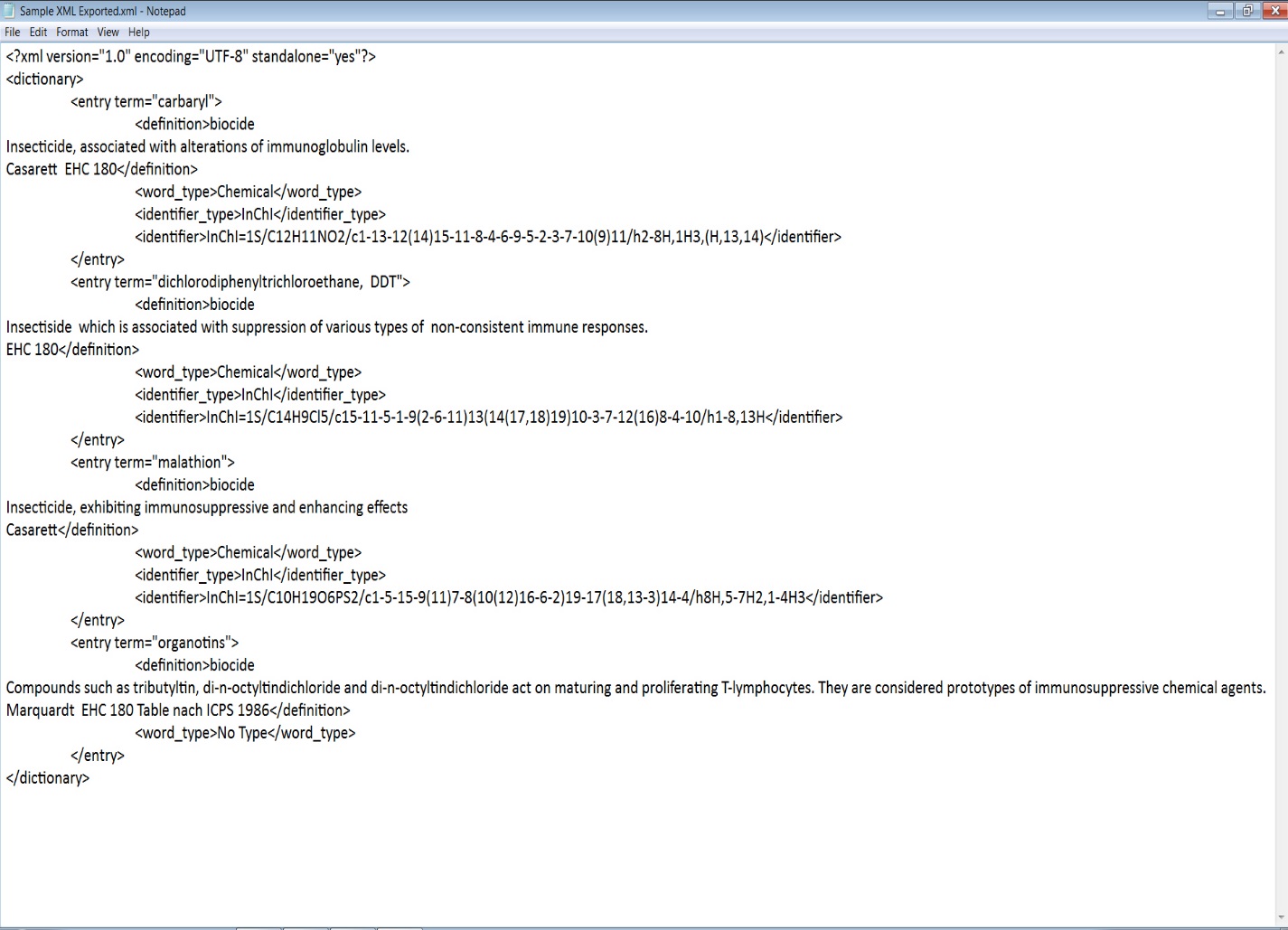


Figure 8: This is a sample glossary in the final XML formatting that the WikiHyperGlossary recognizes and uses for the bulk upload feature to quickly add an entire glossary to the system.

1. Templeton DM, Schwenk M, Klein R, Duffus JH, IUPAC Glossary of terms used in immunotoxicology (IUPAC Recommendations 2012), Pure Appl. Chem., Vol. 84, No. 5, pp.1113-1295, 2012. [Online; accessed: 2015-02-16]. [http://dx.doi.org/10.1351/PAC-REC-11-06-03]
